# Involvement of B2 Receptor in Bradykinin-Induced Proliferation and Proinflammatory Effects in Human Nasal Mucosa-Derived Fibroblasts Isolated from Chronic Rhinosinusitis Patients

**DOI:** 10.1371/journal.pone.0126853

**Published:** 2015-05-13

**Authors:** Yih-Jeng Tsai, Sheng-Po Hao, Chih-Li Chen, Brian J. Lin, Wen-Bin Wu

**Affiliations:** 1 Department of Otolaryngology Head and Neck Surgery, Shin Kong Wu Ho-Su Memorial Hospital, Taipei, Taiwan; 2 Department of Chemistry, Graduate Institute of Applied Science and Engineering, College of Science and Engineering, Fu-Jen Catholic University, New Taipei City, Taiwan; 3 Graduate Institute of Basic Medicine, Fu-Jen Catholic University, New Taipei City, Taiwan; 4 School of Medicine, Fu-Jen Catholic University, New Taipei City, Taiwan; 5 Ronald O. Perelman Department of Emergency Medicine, New York University School of Medicine, New York, United States of America; Chang Gung University, TAIWAN

## Abstract

Chronic rhinosinusitis (CRS) is a chronic inflammatory disease of the sinonasal mucosa either accompanied by polyp formation (CRSwNP) or without polyps (CRSsNP). CRSsNP accounts for the majority of CRS cases and is characterized by fibrosis and neutrophilic inflammation. However, the pathogenesis of CRS, especially CRSsNP, remains unclear. Immunohistochemistry of CRSsNP specimens in the present study showed that the submucosa, perivascular areas, and the mucous glands were abundant in fibroblasts. Therefore, we investigated the effects bradykinin (BK), an autacoid known to participate in inflammation, on human CRSsNP nasal mucosa-derived fibroblasts (NMDFs). BK increased CXCL1 and -8 secretion and mRNA expression with EC_50_ ranging from 0.15~0.35 μM. Moreover, BK enhanced cell proliferation and upregulated the expressions of proinflammatory molecules, including cell adhesion molecules (CAMs) and cyclooxygenase (COX)-1 and -2. These functionally caused an increase in monocyte adhesion to fibroblast monolayer. Using pharmacological intervention and BKR siRNA knockdown, we demonstrated that the BK-induced CXCL chemokine release, cell proliferation and COX and CAM expressions were mainly through the B2 receptor (B2R). Accordingly, the B2R was preferentially expressed in the NMDFs than B1R. The B2R was highly expressed in the CRSsNP than the control specimens, while the B1R and kininogen (KNG)/BK expression slightly increased in the CRSsNP mucosa. Collectively, we report here for the first time that fibroblasts, KNG/BK, and BKRs are overexpressed in CRSsNP mucosa and BK upregulates chemokine expression, proliferation, and proinflammatory molecule expression in NMDFs via B2R activation, which lead to a functional increase in monocyte-fibroblast interaction. Our findings reveal a critical role of fibroblast, KNG/BK, and BKRs in the development of CRSsNP.

## Introduction

Chronic rhinosinusitis (CRS) is characterized by long-term sinonasal mucosa inflammation caused by a large number of cytokines and mediators. The disease has a high prevalence, affecting up to 19.7% of the population in Europe [1] and up to 12.5% in the global population. It results in a substantial burden in terms of health, quality of life, and economical expenditure [2–3]. Despite great advances in the elucidation of its pathophysiology, the exact etiology of the chronic inflammatory conditions of the nose and sinuses is still largely unknown. Based on its tissue remodeling characteristics, CRS can be classified as CRS with nasal polyps (CRSwNP), which is characterized by pseudocyst formation, or without nasal polyps (CRSsNP), which is characterized by the excessive deposition of collagen by the nasal mucosa [4–5]. CRSsNP accounts for the majority of CRS cases (~60%), whereas CRSwNP accounts for 20–33% of cases [6].

Bradykinin (BK) is a potent inflammatory mediator; its signals are mediated via specific cell membrane-anchored G protein-coupled receptors. Two mammalian BK receptor (BKR) subtypes, B1 and B2 receptor (B1R and B2R), have been reported [7]. Immunohistochemical studies of normal and allergic nasal mucosa, epithelial cells, submucosal glands, fibroblasts, macrophages, vascular smooth muscle and endothelial cells showed immunoreactivity for both B1R and B2R [8]. An earlier study showed that kinins are generated *in vivo* following nasal airway challenge of allergic individuals with allergen [9]. In addition, *in vivo* bradykinin nasal challenge causes a significant increase in CXCR1 and CXCR2 mRNA expression in patients with quiescent allergic rhinitis but had no effect in healthy control subjects [10].

CRSwNP and CRSsNP, asthma, and chronic obstructive pulmonary disease (COPD) are similarly characterized by mucosal inflammation and tissue remodeling. Regarding the inflammation, accumulating evidence has shown that chemokine levels play important roles in the progression of airway inflammation [11–12]. Among these chemokines, the CXC chemokines such as CXCL1 and CXCL8, are primarily chemotactic factors for leukocytes and are potent promoters of angiogenesis and tissue inflammation [13–14]. CXCL1 and -8 can bind their receptors, including CXCR1 and -2 [15] and direct the migration of circulating leukocytes to sites of inflammation or injury [16].

The role of BK in fibroblasts in lower, but not in upper airway diseases has been studied. In human lung/bronchial fibroblasts, BK stimulates the expressions of interleukin-1 (IL-1) [17], IL-6, IL-8 [18–19] and eotaxin [20]. In addition, tumor necrosis factor-α (TNF-α) and IL-1β induce an increase in B1R and B2R expressions [21]. Although chronic diseases of upper and lower airway may be associated with similar triggers, the role of inflammatory mediators in the chronic diseases of upper airway remains to be clarified. For example, TGF-β1 has been demonstrated to be up-regulated in CRSsNP and COPD, up-regulated or unchanged in asthma, but down-regulated in CRSwNP [22]. CRSwNP is characterized by edema and eosinophilic inflammation, whereas CRSsNP is characterized by fibrosis and neutrophilic inflammation [1]. Thus, the stromal fibroblasts are hypothesized to be a key player in the pathogenesis of CRSsNP, acting like myofibroblasts (activated fibroblast phenotype) in cardiac and liver fibrosis [23–24].

In this study, CRSsNP mucosa specimens were examined and found to be abundant in stromal fibroblasts as compared to control specimens. Therefore, nasal mucosa-derived fibroblasts (NMDFs) from CRSsNP patients were used as a cell culture model to evaluate the effects of BK on cell proliferation and inflammation. We found that BK induced CXC chemokine release, cellular adhesion molecule (CAM), cyclooxygenase (COX) expression and cell proliferation in the NMDFs via the B2R activation. In accordance with these findings, the nasal mucosa specimens from CRSsNP patients expressed relatively higher B2R and slightly higher kininogen (KNG)/BK and B1R, indicating a role of nasal stromal fibroblast, BK and BKR in pathogenesis of CRSsNP.

## Materials and Methods

### Materials

Bradykinin and 3-[4,5-dimethylthiazol-2-yl]-2,5-diphenyltetrazolium bromide (MTT) were purchased from Sigma (St Louis, MO, USA). HOE140 and R715 were obtained from Tocris Cookson Ltd. (Bristol, BS, UK). The antibodies (Abs) for detecting intercellular adhesion molecule-1 (ICAM-1) and vascular cell adhesion molecule-1 (VCAM-1) were purchased from Cell Signaling Technology (Danvers, MA, USA). The Ab raised against COX-1 was purchased from Santa Cruz Biotechnology (Santa Cruz, CA, USA). The Ab raised against COX-2 was purchased from BD Biosciences (Franklin Lakes, NJ, USA). The Ab against α-tubulin was purchased from Calbiochem EMD Bioscience (San Diego, CA, USA).

### Patient recruitment

This study was approved by the Ethics Committee of the Shin Kong Wu Ho-Su Memorial Hospital, Taipei, Taiwan and conducted with the written informed consent of the patients (Permission no: 20120708R). A total of 12 patients with CRSsNP were recruited and 10 patients who came into correct nasal septal deviations were recruited as a control group. CRSsNP was diagnosed based on patient history and the findings from anterior rhinoscopy, nasal endoscopy, and sinus computed tomography. None of the patients had a history of allergy, asthma, or aspirin sensitivity, and none had been treated with oral or topical anti-allergic agent or steroid for at least 2 months. In the CRSsNP group, the ethmoidal mucosa and the mucosa around the osteomeatal complex were collected during functional endoscopic sinus surgery, and the inferior turbinate mucosae were collected during septoplasty in the control group.

### Isolation of NMDFs

NMDFs were derived from the nasal mucosa of patient with CRSsNP as previously described [25]. Briefly, fragments of the nasal tissues were placed in 6-well culture dishes in DMEM supplemented with 10% fetal bovine serum (FBS) and 100 U/ml penicillin, 100 μg/ml streptomycin, and 2 μg/ml amphotericin B, in a humidified atmosphere at 37°C and 5% CO_2_. Fibroblasts were isolated from the tissue fragments (approximately 3 × 3 mm) through adhesion and migration on a plastic surface, and further characterized by immunostaining of vimentin (Santa Cruz Biotechnology).

### Expression patterns of fibroblasts, BKRs, and kininogen (KNG)/BK in control and CRS biopsies

Immunohistochemistry was performed to identify fibroblasts and determine B1R, B2R and KNG/BK expression in the control and CRS biopsied tissues. Briefly, tissue sections were deparaffinized, and the slides were hydrated through graded ethanol before use. The sections were then washed in TBS (Tris-buffered saline containing 1% CaCl_2_), immersed in sodium citrate buffer (pH 6.0), and heated on a water bath for 20 min. After blocking with buffer containing 10% FBS, the slides were incubated at 4^°^C overnight with primary Ab specific for fibroblasts (clone TE-7, mouse monoclonal IgG1, Millipore, Billerica, MA, USA), B1R, B2R (Abcam) or KNG/BK (LifeSpan BioSciences, Inc., Seattle, WA, USA). Secondary Abs were used at a dilution of 1:250. After additional washing, the slides were stained with one-step 3-amino-9-ethylcarbazole (AEC; Biogenex) for 5–30 min. Sections were counterstained in hematoxylin for 20–40s, washed with tap water, and mounted with 100% glycerol. At least 10–20 random areas of each stained sample from three to five CRSsNP patients or control were analyzed under microscope at magnification of 200X and scored 1~5 based on its overall staining intensity. The representative staining areas scored 1~5 were taken as a reference (S1 Fig).

### Immunocytochemistry of NMDFs

To characterize NMDFs and BKRs expression in NMDFs, cells were seeded on chamber slides. After reaching 80% confluence, cells were washed, fixed with 1% paraformaldehyde (PAF) for 20 min, and permeabilized with 0.1% Triton X-100 for 10 min. Cells were blocked with 3% BSA and followed by incubation with the Abs specific for vimentin (1:200; Santa Cruz Biotechnology), B1R or B2R (1:100, Abcam, Cambridge, MA, USA). After a brief wash, the chamber slides were analyzed under a Nikon Eclipse Ti-S fluorescence microscope (Japan) and photographed using a digital camera.

### ELISA measurement of CXCL1 and -8 secreted in culture medium

The secretion of CXCL1 and -8 in culture medium was determined using the human CXCL1 and -8 ELISA Development kit (R&D Systems, Inc., MN, USA), according to the protocol provided by manufacturer. Briefly, the NMDFs were treated with vehicle or BK. The culture medium was collected and centrifuged, and the secreted CXCL1 and -8 in the culture medium was measured at 450 nm. The absolute concentration of CXCL1 and -8 in the NMDF culture medium was calculated from a standard curve.

### Reverse transcription-polymerase chain reaction (RT-PCR) analysis of CXCL1/8, and BKR mRNA expression

Oligonucleotide PCR primers targeting human CXCL1/8, β-actin, B1R and B2R were synthesized by MDBio Inc. (Taipei, Taiwan; Table 1). Total RNA was extracted using Trizol reagent (Invitrogen Technologies, Carlsbad, CA, USA), and reverse transcription reaction was performed using Superscript III First-Strand Synthesis System (Invitrogen Technologies). RT-PCR analyses of CXC chemokines and β-actin were performed as previously described [26] and the products were revealed by electrophoresis in 2% agarose gel.

**Table 1:** Primers for RT-PCR analysis.

*Gene*	*Forward primer (5’-3’)*	*Reverse primer (5’-3’)*
*B1R*	CTGCACAGAGTGCTGCCAACATT	ACACCAGATCAGAGGCTGCCAGG
*B2R*	CACGGTGCTAGTCCTGGTTGTGCT	AGGTCCGCAGTGTGCCCATG
*β-actin*	ATCATGTTTGAGACCTTCAA	CATCTCTTGCTCGAAGTCCA
*cxcl1*	CATCTCTTGCTCGAAGTCCA	ATCCGCCAGCCTCTATCACA
*cxcl8*	TTTGC CAAGG AGTGC TAAAG	GCATC TGGCA ACCCT ACAAC

### Western blot analysis of CAM and COX expression

Cell lysates were prepared as previously described [27]. Total protein was separated by electrophoresis on SDS-polyacrylamide gels, electroblotted onto PVDF membranes, and then probed using the indicated primary Abs. Immunoblots were developed using Immobilon Western Chemiluminescent HRP Substrate (EMD Millipore).

### Cell growth assay

Cell growth was determined by MTT assay and flow cytometry. For the MTT assay, cells were incubated with 0.5 mg/ml of MTT for 2 h at 37^°^C. Formazan crystals resulting from MTT reduction were dissolved by adding 200 μl of DMSO and gently agitated for 20 min. The absorbance of the supernatant was then measured spectrophotometrically in an ELISA reader at 550 nm. For cell counting by flow cytometry, cells were trypsinized, collected and resuspended in 500-μl serum-free medium. Cells were then analyzed immediately by BD Accuri C6 flow cytometer (Becton, Dickinson and Company, Franklin Lakes, NJ, USA) using forward scatter light (FSC) and side scatter light (SSC) parameters. Number of cells counted in 200-μl medium multiply 2.5 was used to estimate the total cell number in a well.

### Determination of monocyte adhesion to NMDFs

Monocytes were seeded onto 24-well culture plates with or without NMDFs. After reaching confluence, control well (without cells) and confluent cells were incubated with vehicle or 1μM BK for 16 h. Monocytes were labeled with 10 μg/ml of BCECF/AM at 37°C for 30 min and 1 x 10^5^ monocytes were added to plates for adhesion for 1 h. After a brief wash to remove non-adhered monocytes, cell adhesion was measured by direct counting adhered monocytes (as a green spot in the high-power field) in photographs taken randomly by a Nikon Eclipse Ti-S fluorescence microscope (Japan) equipped with a digital camera.

### Measurement of CAM expression on cell surface

NMDFs in monolayer culture were washed with PBS, trypsinized, and collected by centrifugation. The cells were then labeled with FITC- or PE-conjugated control isotype IgG or anti-ICAM-1 or anti-VCAM-1 Abs (1:100, BD Biosciences) in the presence of 1%BSA for 0.5 h at RT with continuous shaking and then analyzed immediately by BD Accuri C6 Flow cytometer. The fluorescence signals from 10,000 cells were collected to calculate mean fluorescence intensity of a single cell.

### B1R and B2R siRNA transfection

ON-TARGET plus SMARTpool siRNA for control, B1R and B2R (Gene ID: NM_000710 for B1R and NM_000623 for B2R) were purchased from Dharmacon RNAi Technologies (Thermo Fisher Scientific, Waltham, MA, USA). NMDFs were seeded in 6- or 24-well plates incubated overnight in complete medium and were transfected with control, B1R or B2R siRNA (150 nM) using the DharmaFECT transfection reagent and cultured for three days. After cells were treated with vehicle or BK, the media were collected for measuring the levels of chemokines by ELISA and the cells were assayed for cell proliferation or harvested for western blotting and RT-PCR analysis.

### Statistical analysis

The data are expressed as the mean ± standard error mean (SEM). A comparison of the means of two groups of data was performed using the unpaired, two-tailed, Student’s *t* test.

## Results

### Determination of fibroblast distribution in control and CRSsNP specimens and characterization of fibroblast culture

**Fig 1:**
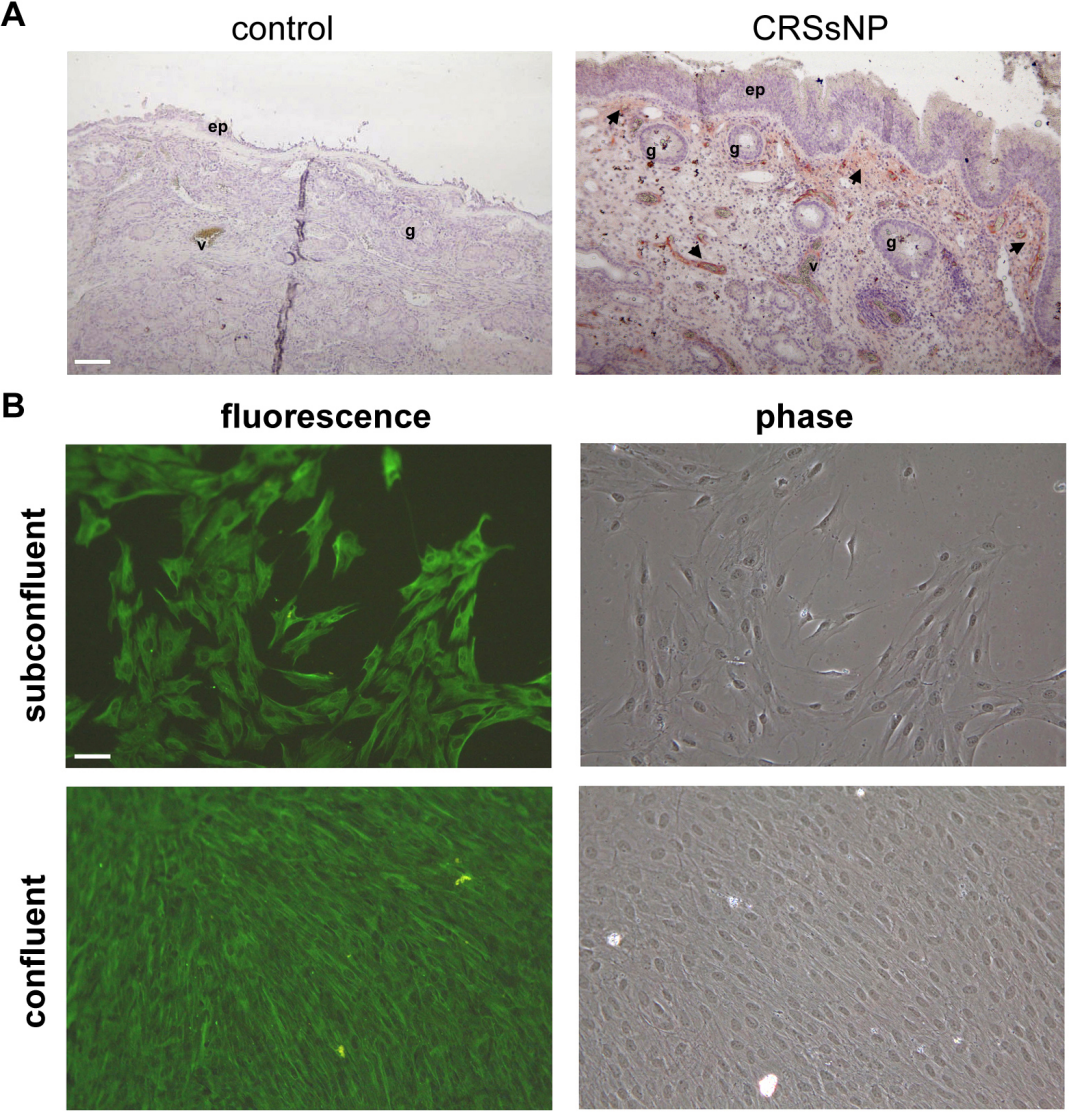
Fibroblast distribution in mucosa specimens and characterization of NMDFs. (A) Immunohistochemical staining showing fibroblast distribution in the normal and CRSsNP nasal mucosa. Inferior turbinates (control) and CRSsNP nasal mucosa were stained with anti-fibroblast Ab. Please note that immunoreactivity for fibroblasts was stronger in the submucosal stroma of CRSsNP specimens than in the nasal septal deviation controls. The arrow indicates a positive staining for fibroblast. ep: epithelial cells; v: blood vessels; g: submucosal glands. Scale bar = 100 μm. (B) Immunocytochemical staining of primary cultured NMDFs. Cells grown on chamber slides in subconfluent and confluent conditions were fixed, stained with anti-vimentin Ab, and analyzed under phase-contrast and fluorescence microscopy. This is representative of three similar experiments.

To investigate whether there is difference in fibroblast distribution in control and CRSsNP nasal mucosa, immunohistochemistry was performed. Fig 1A shows the immunohistochemical staining by the Ab specific for fibroblasts. In the control group, the mucosa showed relatively weak staining reactivity for the Ab. Some of fibroblasts were detected in sub-epithelial region (left panel, very weak deep-red color staining). However, a significant number of fibroblasts could be identified in the submucosal stroma of the CRSsNP specimens. They were noted in the submucosa and perivascular areas (right panel, deep-red color staining).

Next, the primary cultured fibroblasts were prepared from CRSsNP mucosa and the identity was characterized by anti-vimentin Ab. As shown in Fig 1B, the subconfluent and confluent cultured cells were analyzed by phase-contrast microscopy (right panels), showing a typical morphology of fibroblasts. Moreover, the cytoplasm, but not the nucleus, of these cultured cells show strong fluorescence reactivity to anti-vimentin Ab (left panels) by fluorescence microscopy, indicating they were fibroblasts.

### Effects of BK on CXCL1 and -8 protein secretion and mRNA levels

**Fig 2:**
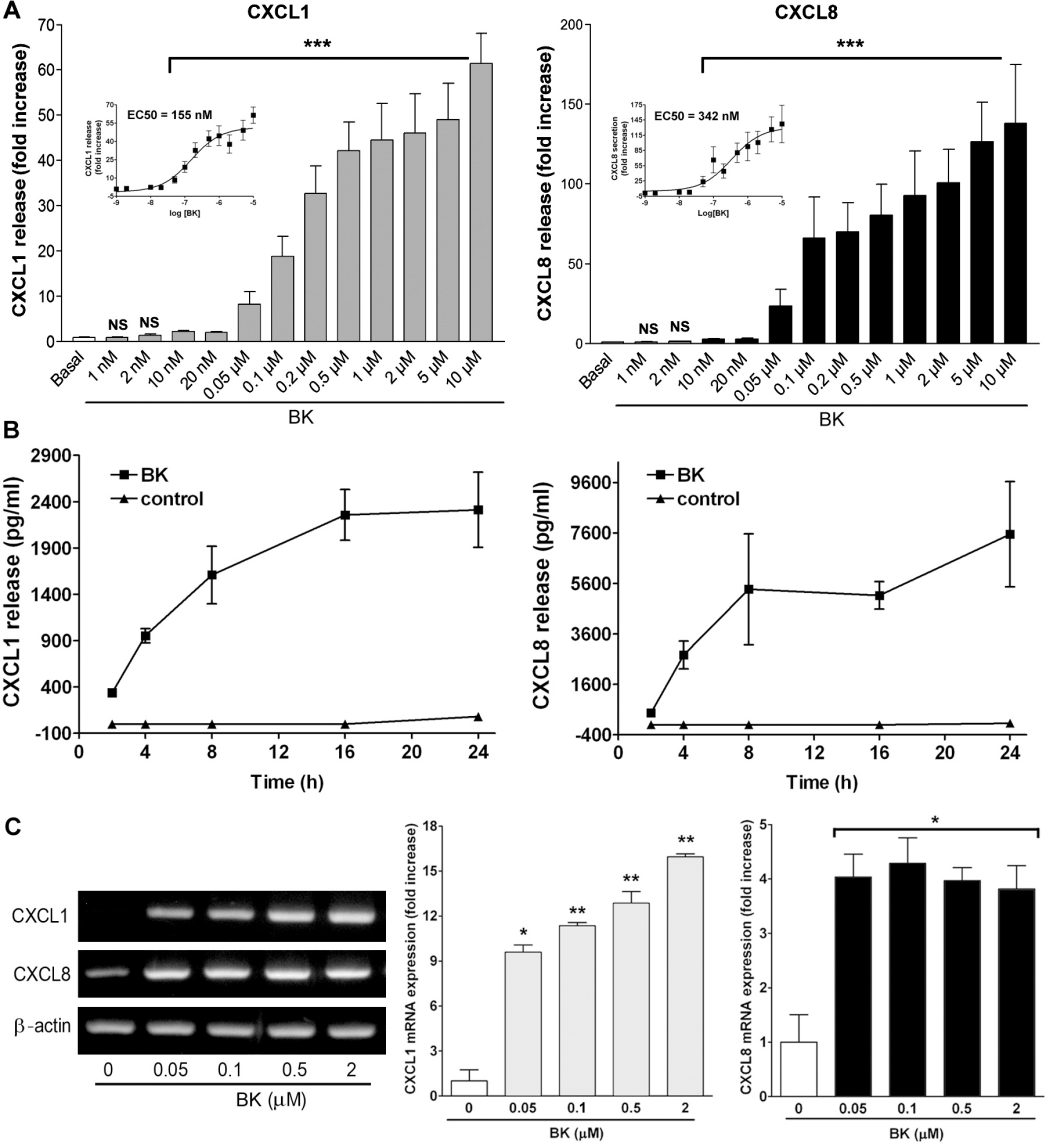
Effects of BK on CXCL1 and -8 secretion and mRNA expression. NMDFs were treated with (A) PBS or the indicated concentrations of BK for 16 h or (B) PBS or BK (2 μM) for the indicated time intervals. CXCL1 and -8 in the culture medium were analyzed by ELISA. The data are mean ± SEM (n = 4–7). Insets in (A): a sigmoidal dose-response curve for calculating EC_50_ of BK in causing CXCL1 and -8 secretion. (C) NMDFs were treated with BK for 6 h. At the end of the incubation, total RNA was extracted, and the expression of CXC chemokines and β-actin mRNA was analyzed by RT-PCR. Data from similar experiments were quantified by densitometry (n = 3). ^*^ *P*<0.05, ^**^ *P*<0.01 versus basal (BK 0 μM).

The concentration- and time-dependent effects of BK on CXCL1 and -8 secretion were examined in NMDFs. As shown in Fig 2A, BK enhanced CXCL1 and -8 secretions in a concentration-dependent manner, with 10nM BK being sufficient to cause the secretion of CXCL1 and -8. The EC_50_ for causing CXCL1 and -8 secretions by BK were estimated to be 155 and 342 nM, respectively (insets). BK also induced CXCL1 and -8 release in a time-dependent manner, and a slight increase was observed at 2-h incubation and a significant increase could be observed after short (4-h) incubation (Fig 2B).

To further determine if BK induces CXCL1 and -8 mRNA expression, NMDFs were treated with BK and after which the expression of CXCL1, -8, and β-actin mRNA was determined by RT-PCR. As shown in Fig 2C, CXCL1 mRNA expression was upregulated by BK in a concentration-dependent manner, whereas CXCL8 mRNA expression was increased by 0.05 μM of BK and appeared to be constant at the following concentrations; however, β-actin mRNA expression remained unaffected. This indicates that BK might affect the levels of CXCL1 and -8 through transcriptional regulation.

### Effects of BK on cell proliferation, proinflammatory molecule expression, and monocyte adhesion

**Fig 3:**
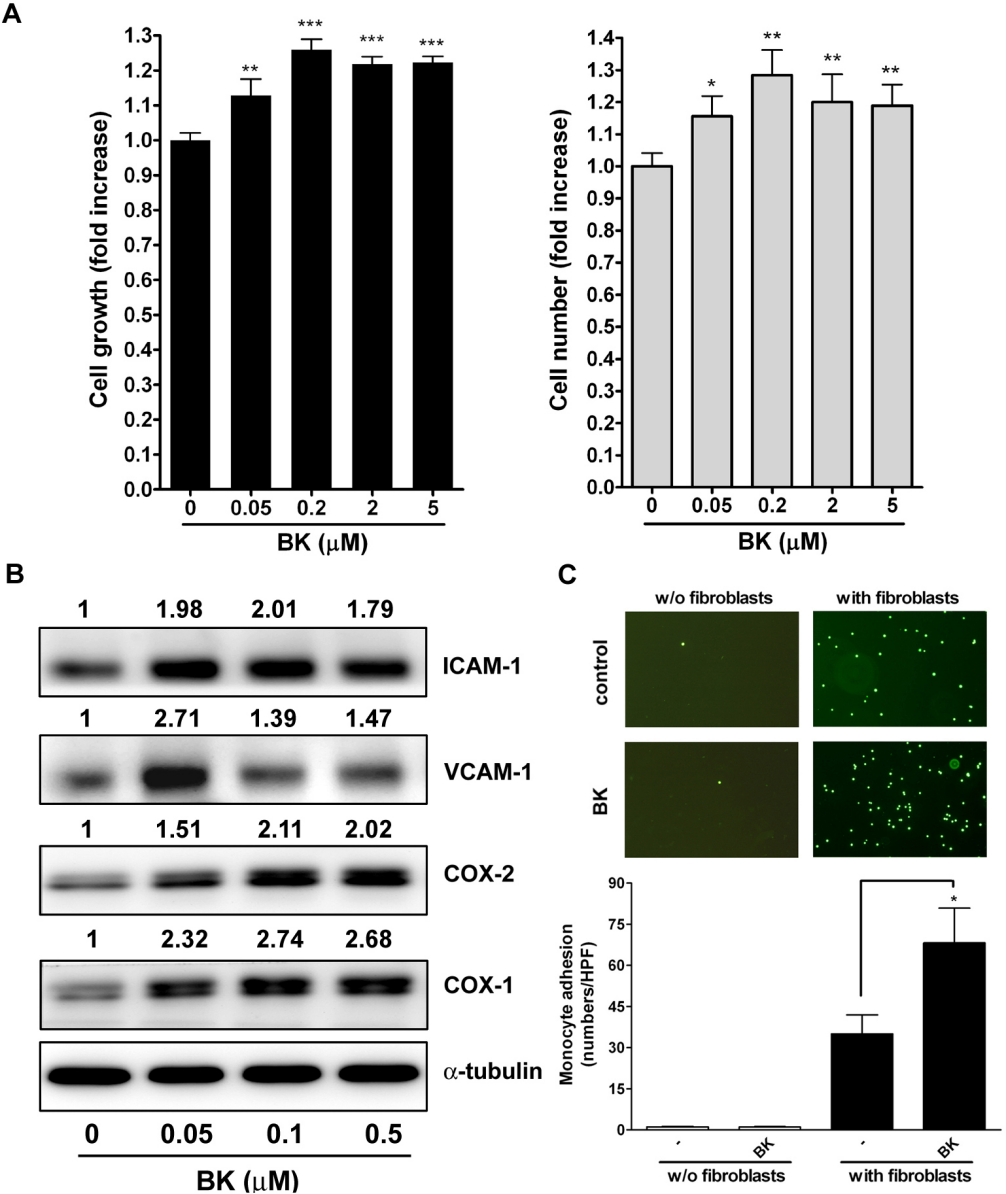
Effects of BK on cell proliferation, proinflammatory molecule expression, and monocyte adhesion. NMDFs were treated with the indicated concentrations of BK for 16 h, and (A) cell proliferation was determined by the MTT assay and direct cell counting assay (n = 3–4). (B) The expression of CAMs and COXs was determined by western blotting (n = 4). (C) Effect of BK on monocytes adhesion to fibroblasts. Twenty-four well plates seeded with or without NMDFs were treated with BK (1 μM) for 16 h. Then, BCECF/AM-labeled monocytes were allowed to adhere to the wells for 1 h. After a brief wash, monocytes adhered to wells (green spots) were determined by direct cell counting under the high-powered field (HPF) (n = 4). ^*^ *P*<0.05, ^**^ *P*<0.01, ^***^ *p* < 0.001 versus basal (BK 0 μM).

To examine whether BK affects fibroblast proliferation, NMDFs were treated with the indicated concentrations of BK for 16 h and cell proliferation was determined by the MTT assay and by direct cell counting using flow cytometry. As shown in Fig 3A,0.05μM BK effectively induced significant cell growth. The cell growth reached a maximum with 0.2 μM of BK (left panel). Flow cytometric analysis of cell number showed similar results to the MTT assay (right panel). Next, NMDFs were treated with the indicated concentrations of BK for 16 h, the expression of CAMs and COXs was then determined by western blot analysis. As shown in Fig 3B, 005μM BK could induce the expression of ICAM-1 and VCAM-1. The VCAM-1 expression by BK was apparent at 0.05 μM of BK but slightly declined when the concentrations increased. Strikingly, BK induced COX-2 and -1 expression in the NMDFs.

The functional consequence of the proinflammatory effects elicited by BK treatment in NMDFs was investigated by performing monocyte adhesion assay. In this regard, BK alone (in the absence of NMDFs) did not cause any monocyte adhesion to plate, indicating that BK did not affect monocyte adhesion function. However, significant monocyte adhesion was noted when monocytes and fibroblasts were cocultured in the presence of BK in the lower chamber, as determined by fluorescence microscopy. This suggests that the augmentation of CXC chemokine secretion and inflammatory molecules expression in NMDFs by BK treatment may enhance monocyte adhesion toward themselves.

### B1 and B2R expression in NMDFs

**Fig 4:**
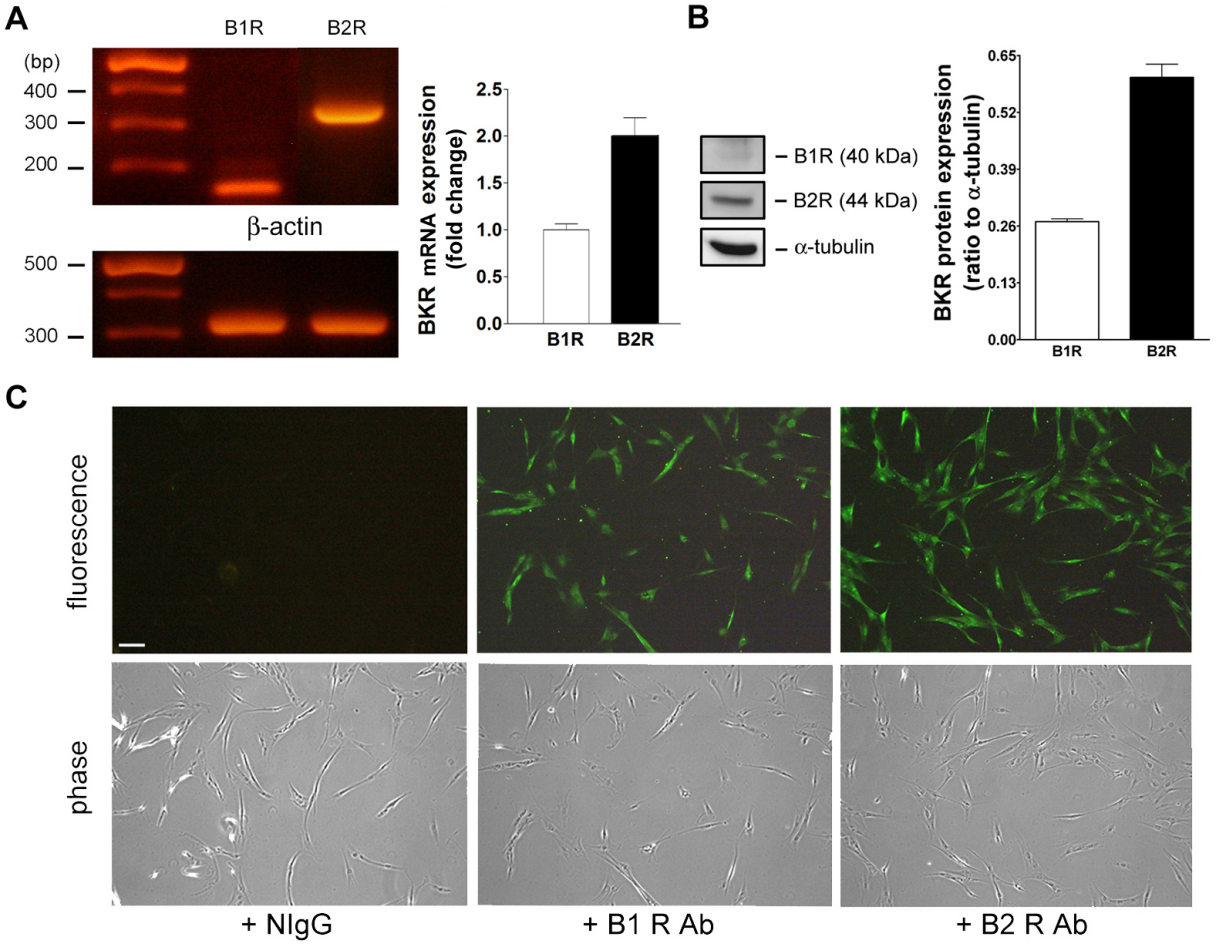
B1 and B2R expression in NMDFs. (A) Total RNA was extracted from NMDFs, and the expression of B1R, B2R, and β-actin was analyzed by RT-PCR and densitometry (n = 3). (B) Western blot analysis of B1 and B2R expression. The B1 and B2R expression in the NMDFs are expressed as ratio of B1 and B2R level to α-tubulin by densitometry, respectively (n = 4). (C) Immunocytochemistry analysis of B1R and B2R expression in NMDFs (n = 3).

We have demonstrated that BK exhibited proinflammatory effects on NMDFs (Figs 2–3). Next, the levels of BKRs on NMDFs were determined. As two mammalian BK receptor subtypes, B1 and B2R, have been reported [7], the expression level of BKRs in the NMDFs was analyzed by RT-PCR. As shown in Fig 4A, B1 and B2R mRNA were expressed in the NMDFs but the B1R was less abundant than the B2R. The differential expression of B1 and B2R was confirmed by western blotting and immunocytochemistry (Fig 4B and 4C). These results indicate that the NMDFs mainly express B2R and less amount of B1R.

### Effect of BKR blockade on CXC chemokine release and fibroblast proliferation

**Fig 5:**
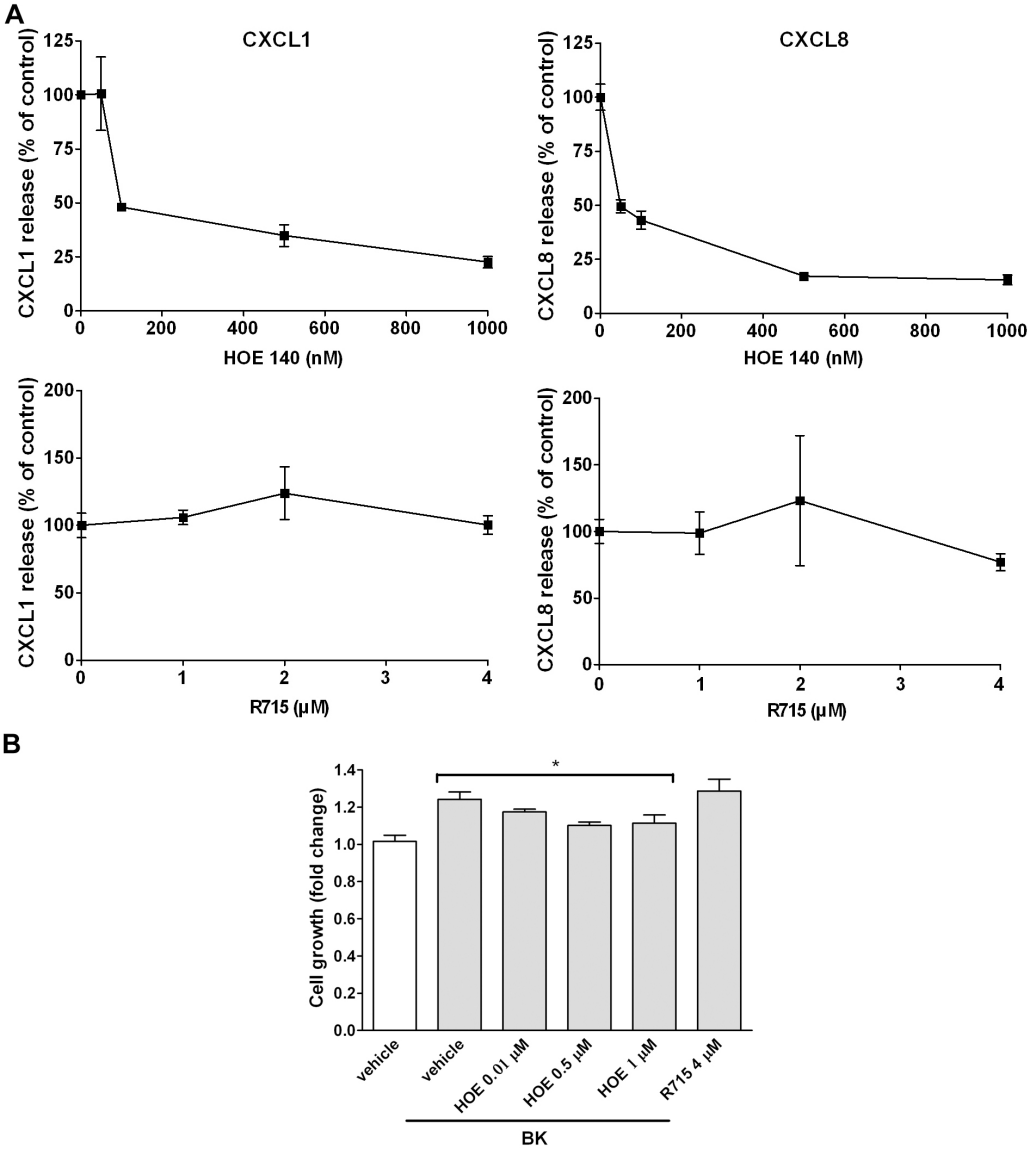
Effects of BKR antagonism on CXCL1 and -8 secretion and cell proliferation. (A) NMDFs were treated with BK in the presence of vehicle or the indicated concentrations of HOE140 or R715 for 16 h, (A) the medium was collected and CXCL1 and -8 secretion was determined by ELISA (n = 3–4) and (B) the remaining adherent cells were determined by the MTT assay to measure cell proliferation (n = 4). ^*^ *P*<0.05 versus BK control.

Next, we examined which receptor is responsible for CXC chemokine release and cell proliferation by BK in NMDFs. As shown in Fig 5A, NMDFs were treated with the indicated concentrations of HOE140 or R715, two selective antagonists for B2R and B1R respectively. After which, the cells were treated with BK and the secretion of CXCL1 and -8 was measured. HOE140 inhibited the secretion of CXCL1 and -8 in a concentration-dependent manner. The inhibition was achieved at 100 nM and almost reached a maximum at 500 nM. However, R715, did not inhibit BK-induced CXCL1 and -8 secretion. Similarly, HOE140 (0.01 μM) was sufficient to inhibit BK-induced cell proliferation, while R715 at a higher concentration (4 μM) did not exhibit any inhibitory effect on BK-induced cell proliferation.

### Effect of BKR antagonism on BK-induced proinflammatory molecule expression

**Fig 6:**
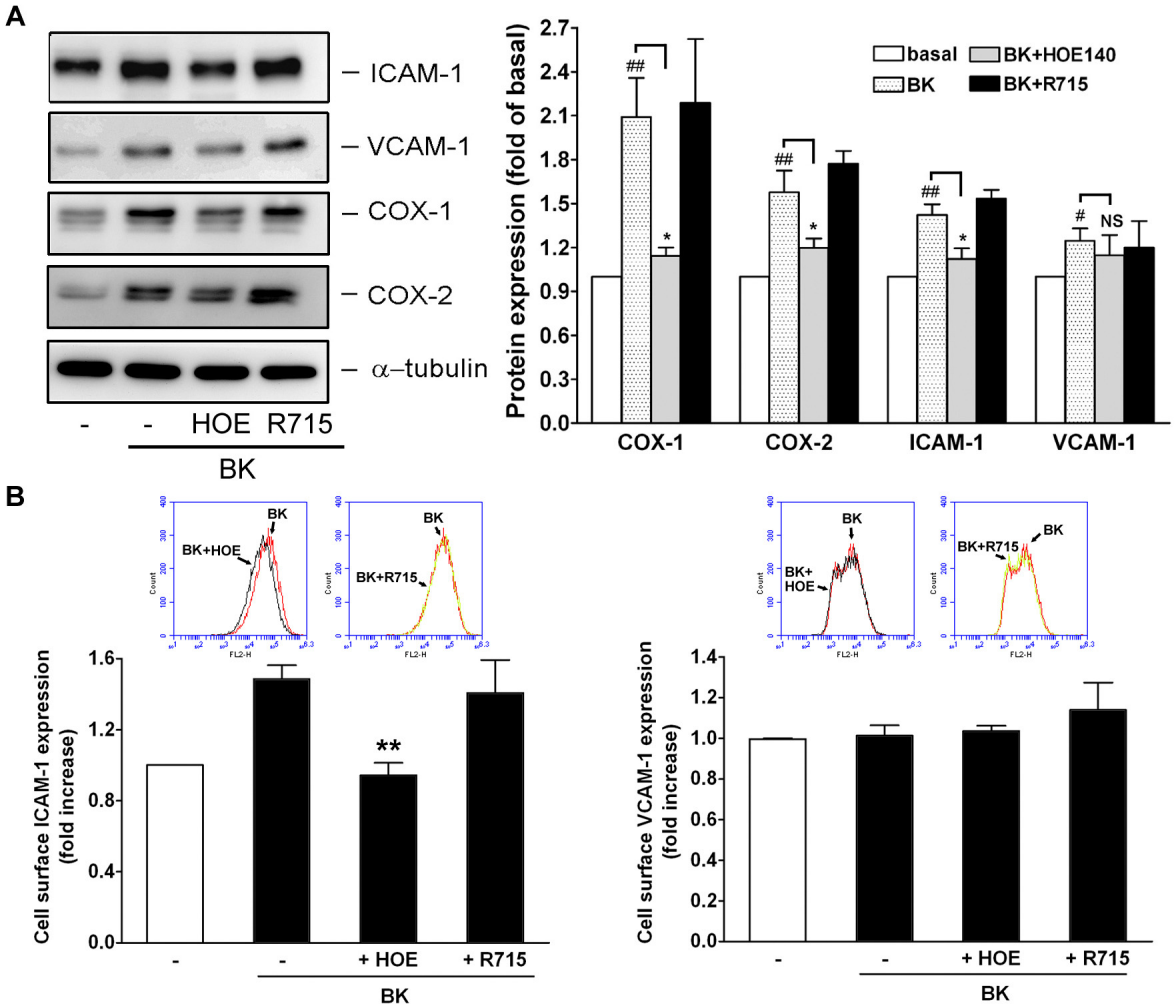
Effects of BKR antagonism on the expression of proinflammatory molecule. The NMDFs were treated with BK (0.5 μM) in the presence of vehicle, HOE140 (0.5 μM) or R715 (2 μM) for 16 h. (A) The expression of CAMs and COXs in the cells was determined by western blotting and quantitated by densitometry (n = 4). (B) The ICAM-1 and VCAM-1 expression on the cell surface was determined by flowcytometry and the representative histograms were shown in the upper panels. Quantitative analysis of mean fluorescence intensity (MF) from similar results are shown in the lower panels (n = 3–5). ^#^ *P*<0.05, ^##^ *p* < 0.01 versus basal; ^*^ *P*<0.05, ^**^ *P*<0.01 versus BK control. NS: non-significant.

The previous data have shown that the levels of COX-1, COX-2, ICAM-1 and VCAM-1 were markedly upregulated after treatment with BK (Fig 3B). Therefore, we examined whether blockade of B1 and B2R affects this induction. As shown in Fig 6, the induction of these proinflammatory molecules, except for VCAM-1 expression, was significantly inhibited by HOE140 but not by R715. Further, flowcytometric analysis indicated that BK-induced ICAM-1 but not VCAM-1 expression on cell surface was significantly reduced by HOE140.

### Effect of BKR KD on BK-induced chemokine release, cell proliferation, and proinflammatory molecule expression

**Fig 7:**
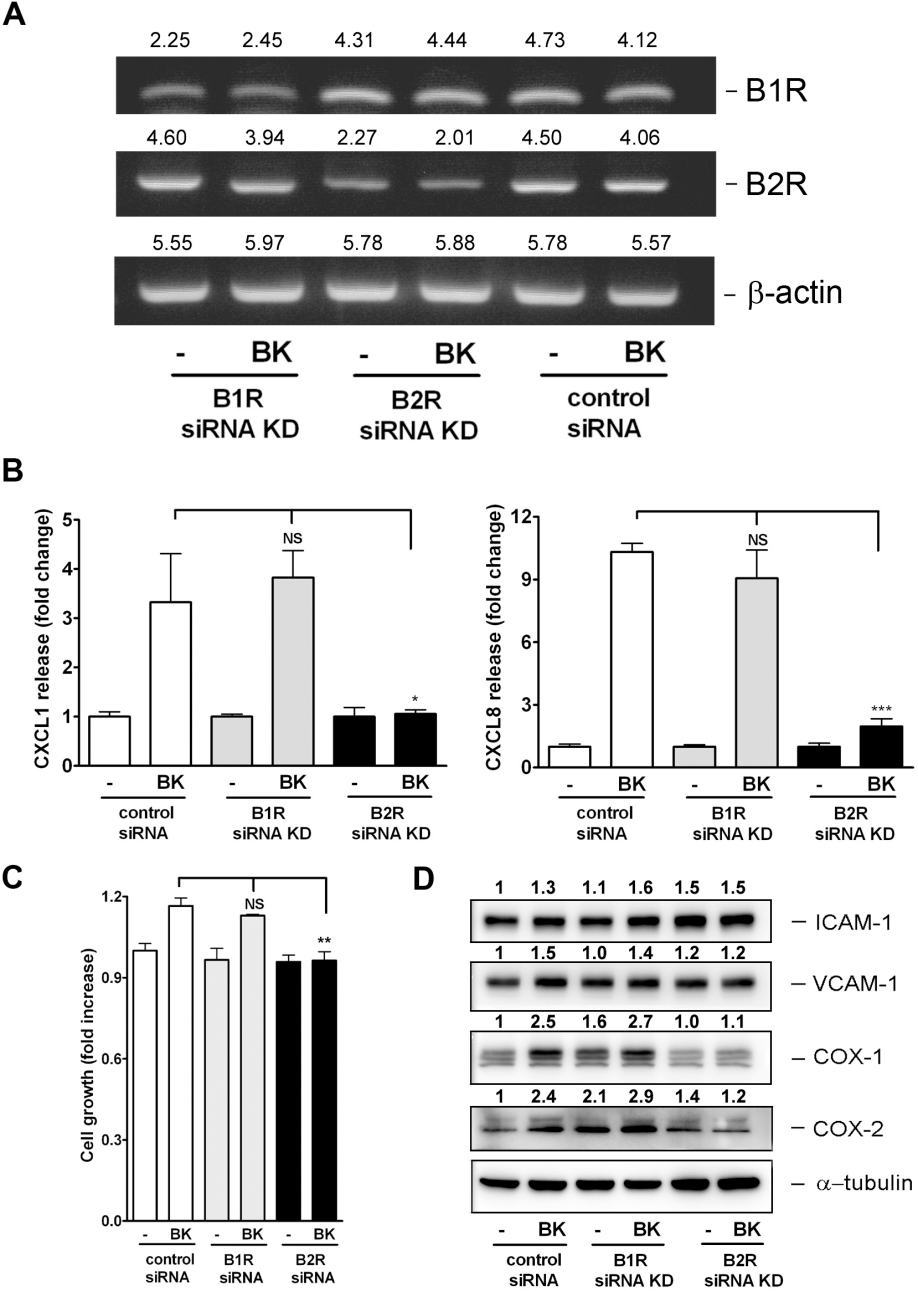
Effects of BKR KD on cell proliferation and proinflammatory molecule expression in NMDFs. Cells were transfected with control, B1R or B2R siRNA. (A) The KD efficiency of each siRNA was examined by analyzing the B1, B2R and β-actin expression with RT-PCR (n = 2). (B) The CXCL1 and -8 secretion in supernatant, (C) cell proliferation, and (D) proinflammatory molecule expression by BK treatment (1 μM) were determined by ELISA, MTT, and western blotting, respectively (n = 3). ^*^ *P*<0.05, ^**^ *P*<0.01 and ^***^ *P*<0.001 versus BK control.

The BK-induced chemokine release and proinflammatory molecule expression could be blocked by the B2R pharmacological inhibitor (Figs 5 and 6). Therefore, SiRNA KD of B1R and B2R was performed to verify the importance of B2R in mediating BK-induced cell proliferation and proinflammatory molecule expression in NMDFs. As shown in Fig 7A, B1 and B2R siRNA KD obviously reduced B1 and B2R expression respectively and the control siRNA had no effect on BKRs expression. Under these conditions, the CXCL1 and -8 release by BK was notably reduced in cells by the B2R KD; however it remained unchanged in cells transfected with the B1R and control siRNA. Consistently, cell proliferation and CAM and COX expressions were suppressed by the B2R but not B1R KD (Fig 7B–7D).

### BKR and KNG/BK expression patterns in mucosa of control and CRSsNP specimens

**Fig 8:**
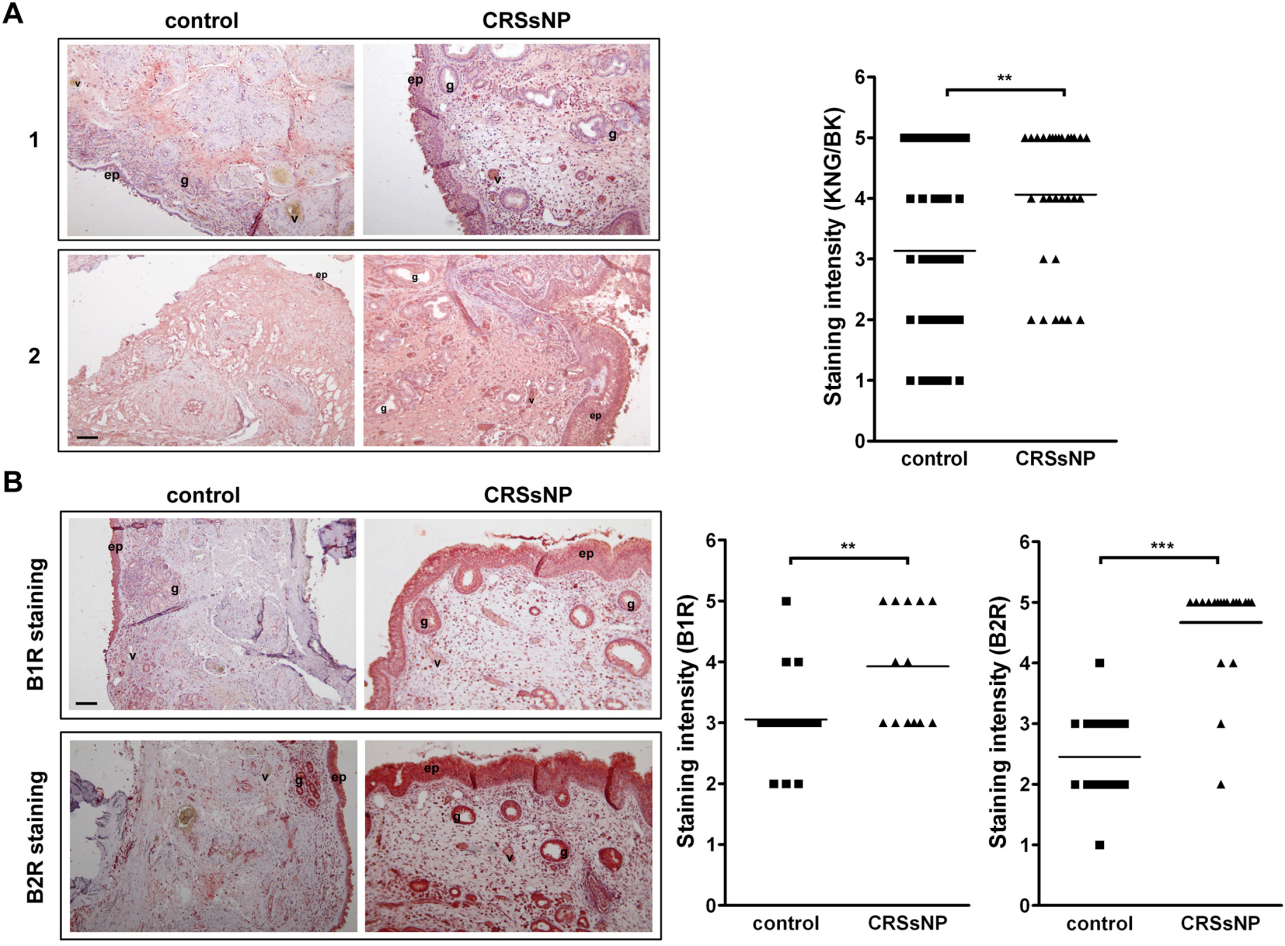
KNG/BK and BKR expression patterns in normal and CRSsNP mucosa. The control and CRSsNP mucosa specimens were stained with (A) anti-KNG/BK or (B) anti-BKR Abs. At least 10–20 random areas of the staining from control and CRSsNP patients (n = 3–5) were scored 1~5 based on the overall staining intensity. ep: epithelial cells; v: blood vessels; g: submucosal glands. Scale bar = 100 μm. ^**^ *P*<0.01, ^***^ *p* < 0.001 versus control.

It has been reported that generation of BK or kallidin (Lys-BK) results from a complex multistep and enzymatic cleavage by kallikrein from KNG [28]. To further investigate whether BK and its precursor (KNG) are expressed in nasal mucosa, immunohistochemistry of the specimens was performed using the Ab raised against KNG and BK. In Fig 8A (left panels), two representative mucosa specimens from different control and CRSsNP patients were shown. The KNG/BK was mainly expressed in the epithelium and submucosal stroma. According to the scatter plot (Fig 8A, right panel), the KNG/BK in control specimens were differentially expressed with staining intensity score from 1 to 5. However, the overall KGN/BK expression appeared to be slightly increased in the mucosa of CRSsNP patients.

As we have shown that BK induced CXC chemokine release and proinflammatory effects in NMDFs, we next investigated the BKR expression patterns in the control and CRSsNP nasal mucosa. As shown in Fig 8B, the B1R was expressed in epithelium and submucosal glands of control nasal mucosa and were also slightly expressed in epithelium, submucosal glands, stromal fibroblasts, and vessel (endothelium) of the CRSsNP mucosa (deep-red color staining). Moreover, the B2R was mainly expressed in the epithelium and submucosal glands and partially expressed in vessels of control nasal mucosa but apparently expressed in epithelium, submucosal glands, stromal fibroblasts, and vessel (endothelium) of CRSsNP mucosa (lower panels). The areas of each stained sample from control and CRSsNP patients were scored. The B1R expression slightly increased, whereas the B2R expression increased more robust in the CRSsNP mucosa specimens. The stromal fibroblasts may be as one of the cell types that highly express B2R.

## Discussion

CRS is an inflammatory condition of the mucosa in the nasal cavity and paranasal sinuses. During CRS development, nasal epithelial cells have been shown to participate in many of the inflammatory processes [29–30]. Along with nasal epithelial cells, sinonasal fibroblasts also participate in these inflammatory processes based on their ability to secrete a variety of cytokines and chemokines in response to proinflammatory cytokines, such as IL-1β and TNF-α [31]. Jung et al. have demonstrated that nasal fibroblasts play a substantial role in airway inflammation, such as virus-induced upper airway inflammation [32]. In accordance with these observations, CRSsNP is characterized by a more neutrophilic inflammation, together with fibrosis formation within the extracellular consisting of excessive collagen deposition and thickening of the collagen fibers [33]. Thus, fibroblasts may be an important target in investigating the pathogenesis of CRSsNP. In this study, we showed that fibroblasts were apparently abundant in the CRSsNP than the control mucosa specimens and were mainly located in the submucosa stroma and perivascular region of the CRSsNP specimens (Fig 1A), suggesting that stromal fibroblasts play a role in the pathogenesis of CRSsNP.

Rudack et al. have shown that both GRO-α (CXCL1) and GCP-2 contribute to neutrophil chemotaxis in CRS, whereas IL-8 (CXCL8) and ENA-78 appear to be of secondary importance for the chemotaxis of neutrophils [14]. In this study, the CXCL1 and -8 secretions were increased following stimulation of NMDFs by BK in a time- and concentration-dependent manner, with the EC_50_ at 155 and 342 nM, respectively. Moreover, BK notably induced CXCL1 and -8 mRNA expression (Fig 2), suggesting that BK functions as an effective and potent stimulator on CXC chemokines release in NMDFs via transcriptional regulation. Moreover, BK caused 1.2~2.1 fold increase in causing proinflammatory molecule expression (Figs 3 and 6), indicating that BK is a moderate inducer on COX and CAM expression. The induction of chemokines and proinflammatory molecules by BK leads to an inflammatory condition of fibroblasts as it enhanced monocyte adhesion (Fig 3C).

BK has been reported to be involved in the proliferation of human bronchial fibroblasts from asthmatics and human normal bronchial fibroblasts during asthma through B2R activation [34]. This process indicates that BK may participate in lower airway remodeling in asthma. We showed here for the first time that BK could induce proliferation in the NMDFs (Fig 3A). The proliferation could be blocked by the B2R antagonism and KD, but not by the B1R antagonism and KD. Moreover, the B2R mediates BK-mediated CXC chemokine release and proinflammatory molecule expression (Figs 5–7). Taken together, our data suggest that B2R is the main receptor responsible for BK-mediated function in NMDFs. Cheng et al. also reported that BK can induce the proliferation of Statens Seruminstitut Rabbit Corneal Cells in a B2R-dependent and EGFR transactivation pathway [35].

There is evidence showing that in the allergic diseases, such as asthma and allergic rhinitis, the major pharmacological effect of kinin is mediated through the B2R pathway [36–37]. However, Christiansen et al. have shown that functional B1R are induced in the airway of patients with allergic rhinitis and this upregulation leads to activation of certain gene transcription [38]. In addition, BK can be recovered in the nasal lavage from patients with allergic rhinitis after allergen provocation and is involved in many nasal symptoms [39–40]. In this study, KNG/BK slightly increased in the CRSsNP than control mucosa (Fig 8A). However, no commercially available Ab can distinguish whether they were KNG or BK because they share consensus amino acid sequences. As BK is generated from enzymatic cleavage by kallikrein from KNG, our data reveal the abundance and availability of KNG/BK both in normal and CRSsNP nasal mucosa. In contrast to the slightly increased expression of KNG/BK and B1R in the CRSsNP mucosa, a higher-level expression of the B2R in nasal epithelial cells, stromal fibroblasts, and glands was observed in the CRSsNP mucosa (Fig 8B). This finding is different from the study that B1 and B2R are about equally expressed in normal nasal mucosa and active allergic rhinitis tissue [8], may account for different pathophysiological phenomena existing between allergic rhinitis and CRSsNP. Consistently, B1R was expressed in a lower level, whereas B2R was highly expressed in the NMDFs (Fig 4). It is suggested that B2Rs are constitutively expressed on many cell types [41], whereas B1Rs expressed at low levels in normal tissues but can be induced in response to pathophysiological stimuli [42]. Indeed, our preliminary result indicates that B1R, but not B2R, could be upregulated in the NMDFs upon lipopolysaccharide (LPS) and TNF treatment (our unpublished data). As LPS and TNF are potent proinflammatory agents found in bacterium and in chronic inflammation conditions, this possibly explains, at least in part, why an increased expression in B1R was observed in CRSsNP mucosa.

An interesting finding in this study is that BK induced both COX-1 and COX-2 expression in the NMDFs (Figs 3B, 6A and 7D). It is known that prostaglandin (PG) production which is mediated by COX commonly plays a substantial role in the inflammation process. However, the regulatory mechanisms of COX remain unclear in pathogenesis of CRSsNP [43–47]. We hypothesize that COX-1 upregulation may lead to generation of some PGs such as PGI_2_ and PGE_2_ to maintain homeostasis, whereas COX-2 upregulation may participate in chronic inflammation. The upregulation of CAMs in cellular lysates by BK stimulation is also an interesting observation. However, only ICAM-1 protein was significantly expressed in cells and on cell surface of the NMDFs challenged with BK (Fig 6B). To our knowledge, there are few reports examining the role of CAM expression by BK in fibroblasts derived from airway and CRSsNP tissues. ICAM-1 is a counter receptor for leukocyte integrin α_L_β_2_ and integrin α_M_β_2_ receptors [48], and can be exploited by rhinovirus as a receptor [49]. Our result suggests the stromal fibroblasts in CRSsNP may act as a functional receptor in the host for interacting with leukocytes and infected viruses.

Several lines of evidence have shown that BK plays important roles in allergic rhinitis [9–10]. Since none of the patients we recruited in this study had a history of allergy or asthma sensitivity, our findings suggest that BK can serve as an important player in CRS and BK and B2R may be taken as a therapeutic target for treating/preventing CRS. Moreover, the abundance of stromal fibroblasts in the CRS biopsied tissue indicates the possible key role in the pathogenesis and pathophysiology of CRSsNP.

In conclusion, we demonstrate here for the first time that fibroblasts are overexpressed in CRSsNP mucosa, mainly located at submucosa stroma, perivascular region, and mucous glands. Moreover, the immunoreactivity for KNG/BK and BKRs increased in the nasal epithelial cells, stromal fibroblasts, and glands of the CRSsNP mucosa. BK upregulates CXCL1 and -8 chemokine mRNA and protein expression, proliferation and proinflammatory molecule expression in CRSsNP-derived fibroblasts through B2R activation. The induction finally leads to an increase in monocyte-fibroblast interaction, revealing an important role of nasal mucosa stromal fibroblasts and BK in CRSsNP development.

## Supporting Information

S1 Fig The representative panel (score 1~5) as a reference for scoring the positive staining areas from control and CRSsNP mucosa specimens. (DOCX)
