# Supplementary material for: Involvement of B2 Receptor in Bradykinin-Induced Proliferation and Proinflammatory Effects in Human Nasal Mucosa-Derived Fibroblasts Isolated from Chronic Rhinosinusitis Patients
Source: PLoS One. 2015 May 13;10(5):e0126853. doi: 10.1371/journal.pone.0126853 (PMC4430235; doi:10.1371/journal.pone.0126853)

**Supporting information**

S1 Fig. The representative panel (score 1~5) as a reference for scoring the positive staining areas from control and CRSsNP mucosa specimens.


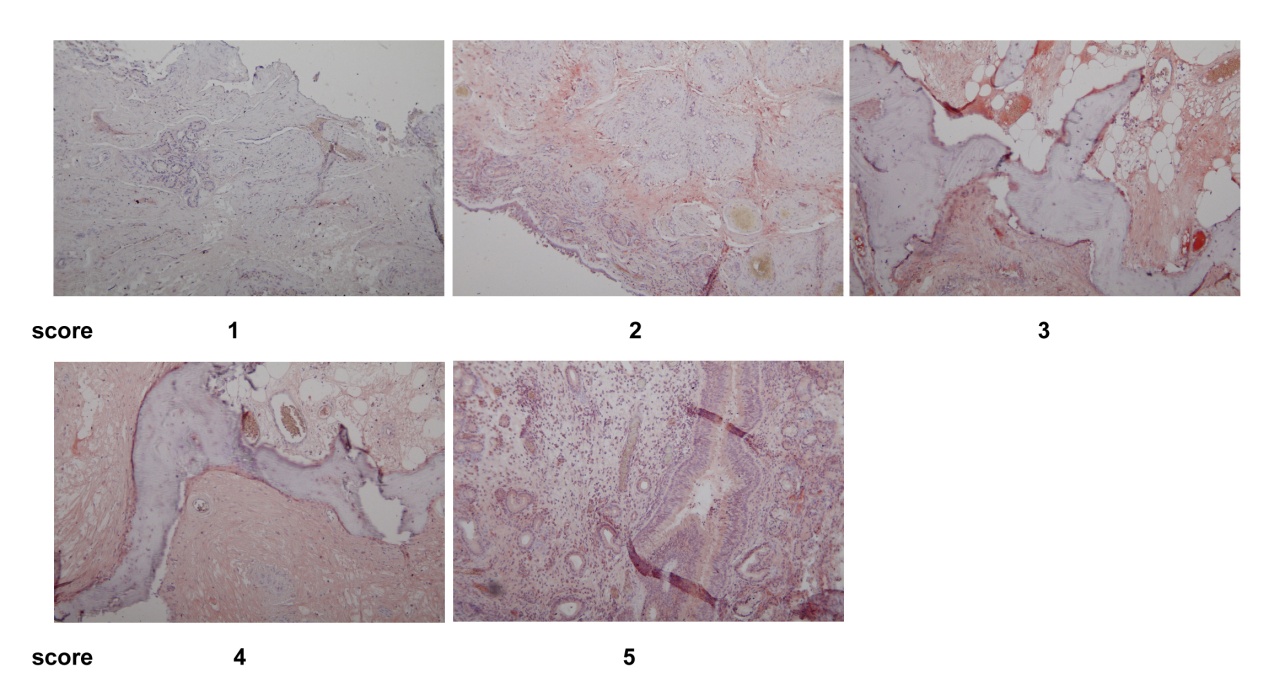

Supplement: S1 Fig — (DOCX) [file pone.0126853.s001.docx]
